# Delayed Inflammatory Reactions to Hyaluronic Acid Fillers in a Patient Undergoing Dupilumab Therapy: A Case Report

**DOI:** 10.1111/jocd.70997

**Published:** 2026-08-07

**Authors:** Osamah Alkhuzaim, Abdullah Almeziny

**Affiliations:** ^1^ College of Medicine Imam Mohammad Ibn Saud Islamic University (IMSIU) Riyadh Saudi Arabia

## Abstract

**Background:**

Delayed inflammatory reactions to hyaluronic acid (HA) dermal fillers are uncommon but clinically significant, particularly in patients receiving immunomodulatory biologic therapies. Dupilumab, an interleukin‐4 receptor alpha (IL‐4Rα) inhibitor approved for chronic rhinosinusitis with nasal polyposis (CRSwNP) and other type 2 inflammatory conditions, has not been previously associated with this adverse event.

**Objective:**

To report a case of delayed inflammatory filler reactions at previously injected hyaluronic acid filler sites associated with dupilumab administration, and to discuss proposed immunological mechanisms, differential diagnoses, and management considerations.

**Case Presentation:**

A 34‐year‐old female with refractory CRSwNP, who had previously tolerated HA skinbooster injections without complications, developed facial warmth, pain, and hyperpigmented swelling at prior filler sites within 1 h of receiving her first dupilumab 600 mg dose. Within 24 h symptoms progressed to persistent bilateral cheek nodules. Similar, although to a lesser extent, reactions were observed after some of the later doses of dupilumab and treatment with hyaluronidase resulted in partial resolution. Reactions reappeared with additional dosing of dupilumab, requiring further doses of hyaluronidase to mitigate these reactions.

**Results:**

Score on the Naranjo Adverse Drug Reaction Probability Scale: 8 (probable but not confirmed temporal correlation between dupilumab and inflammatory filler reaction). One possible mechanistic explanation is that dupilumab‐mediated IL‐4/IL‐13 inhibition may shift ambient filler sites toward Th1/Th17‐dominant immune activity; this hypothesis remains speculative without histopathological confirmation. Alternative diagnoses including infection, biofilm formation, and idiopathic granulomatous inflammation cannot be excluded.

**Conclusion:**

To our knowledge the first case report of delayed inflammatory filler reactions associated with biologic therapy. This potential interaction is important since it may expose patients who are on biologics to increased susceptibility to complications following esthetic procedures, and thus clinicians performing these esthetic treatments in these patients should be aware of this risk. This is constrained by the lack of imaging and biopsy investigation. More studies are necessary for understanding the mechanisms of this association and improving management strategies.

## 1 Introduction

The demand for nonsurgical cosmetic procedures has grown substantially in recent years, driven by advances in technique, product development, and the appeal of minimally invasive treatments [1]. Among these, hyaluronic acid (HA) dermal fillers represent the second most frequently performed nonsurgical esthetic procedure worldwide, offering effective solutions for facial rejuvenation, volume restoration, and skin quality improvement [1]. The overall safety profile is well established, and serious adverse reactions are uncommon.

HA filler products differ considerably in their physicochemical properties, including their degree of cross‐linking, viscosity, and rheological behavior. These properties determine their clinical application. Volumizing fillers, typically high‐viscosity, highly cross‐linked formulations, are injected into the deep dermis or subcutaneous tissue to restore facial volume and projection. In contrast, skinbooster‐type formulations are low‐viscosity, lightly cross‐linked products designed to improve skin hydration, elasticity, and overall skin quality rather than to provide volumization. These are administered via intradermal microdroplet injection in a grid‐like pattern and are not intended for deep structural augmentation [1, 2, 3, 4].

Cross‐linked HA products have been associated with higher incidences of delayed inflammatory reactions compared to NASHA formulations [5]. These reactions are late‐onset immune responses typically mediated by T‐cell lymphocytes that can manifest as tender, erythematous swelling or persistent nodules at injection sites, appearing weeks to months after the original procedure [5].

Chronic rhinosinusitis with nasal polyposis (CRSwNP) is a chronic inflammatory airway condition affecting approximately 4% of the United States population [6]. Dupilumab, a fully human monoclonal antibody targeting the shared IL‐4Rα subunit of the IL‐4 and IL‐13 receptors, is FDA‐approved for CRSwNP, atopic dermatitis, and asthma. It is generally well tolerated, with the most commonly reported adverse effects being transient eosinophilia, ocular surface reactions, and injection‐site discomfort [6]. No prior reports have linked dupilumab to inflammatory reactions at previously injected filler sites.

We report a case of delayed inflammatory filler reactions developing at previously placed HA skinbooster sites following dupilumab administration, discuss the proposed immunological mechanism, and review relevant management considerations. To our knowledge, this is the first published case report documenting a probable association between dupilumab therapy and delayed inflammatory reactions to hyaluronic acid fillers and the first reported case of this phenomenon occurring in the setting of biologic immunomodulatory therapy.

## 2 Case Report

A 34‐year‐old woman with refractory chronic rhinosinusitis with nasal polyps (CRSwNP) presented to our clinic on May 9, 2024, seeking improvement in skin hydration, texture, and overall facial radiance. She had previously undergone several intradermal hyaluronic acid (HA) filler treatments without any adverse events. Treatment was performed using a lightly cross‐linked, low‐viscosity HA skinbooster formulation, which is specifically designed to improve skin hydration, elasticity, and overall skin quality rather than to provide structural volumization. A total of 1 mL was injected into each cheek using an intradermal microdroplet technique. The product was deposited into the deep dermis at an angle of less than 45° to the skin surface, with injection points spaced approximately 0.5–1.0 cm apart across the bilateral malar regions. The procedure was performed under strict aseptic conditions and was completed without complications.

**FIGURE 1:**
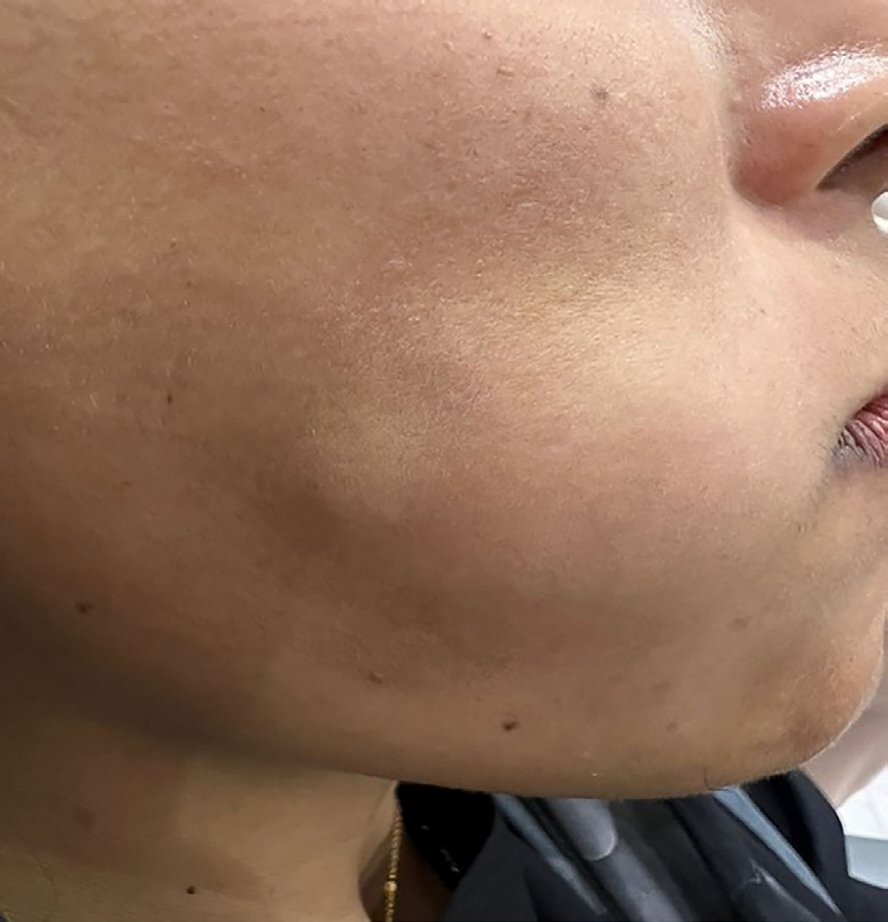
Delayed inflammatory reaction to hyaluronic acid filler associated with dupilumab. Right oblique view of the face shows firm, tender, mildly erythematous nodules in the right submalar region, at sites of prior intradermal hyaluronic acid skinbooster injection.

**FIGURE 2:**
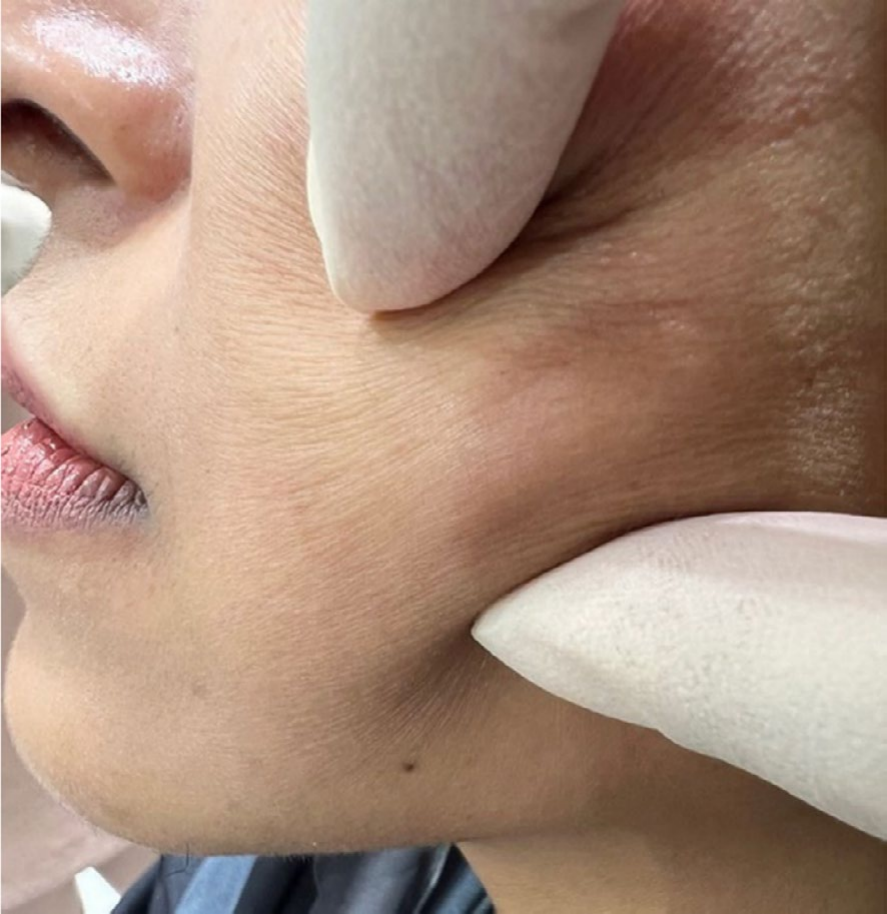
Left oblique view of the same patient at the same visit, showing corresponding firm, tender nodules in the left submalar region at sites of prior hyaluronic acid skinbooster injection.

On August 1, 2024, approximately 1 h after receiving her first loading dose of Dupilumab (600 mg), the patient developed acute facial warmth, pain, and erythematous swelling localized precisely to the sites of her prior HA skinbooster injections. Over the ensuing 24 h, multiple discrete palpable papules and nodules developed at the individual injection points and progressively coalesced into firm, persistent nodules involving both malar regions (Figures 1 and 2). A similar inflammatory reaction recurred following the patient's second maintenance dose of dupilumab (300 mg) on August 25, 2024, although the severity was less pronounced than that observed after the initial loading dose.

On September 3, 2024, the patient presented to our clinic for evaluation of persistent bilateral malar nodules, more prominent on the right side. She did not seek medical attention after the initial episode because the symptoms partially improved without intervention. However, after the reaction recurred following her second dupilumab dose and the nodules persisted and became more conspicuous, she sought further evaluation. Physical examination revealed firm, mildly tender nodules localized to the sites of the previous HA skinbooster injections. There was no overlying erythema, fluctuance, drainage, or evidence of systemic infection, including fever. The clinical impression that the nodules represented an inflammatory reaction to residual filler material, and in the absence of findings suggestive of infection, Hyaluronidase was selected as the initial treatment. The rationale was to enzymatically degrade the residual HA filler presumed to be acting as the antigenic substrate driving the inflammatory response. Systemic corticosteroids were considered but deferred because the diagnosis remained presumptive. Antihistamines were not administered because the presentation was not consistent with an immediate IgE‐mediated hypersensitivity reaction, and antibiotics were withheld because there were no clinical signs of bacterial infection. A total of 150 units of hyaluronidase was injected bilaterally. The patient reported a marked reduction in nodule size within 5 days.

**FIGURE 3:**
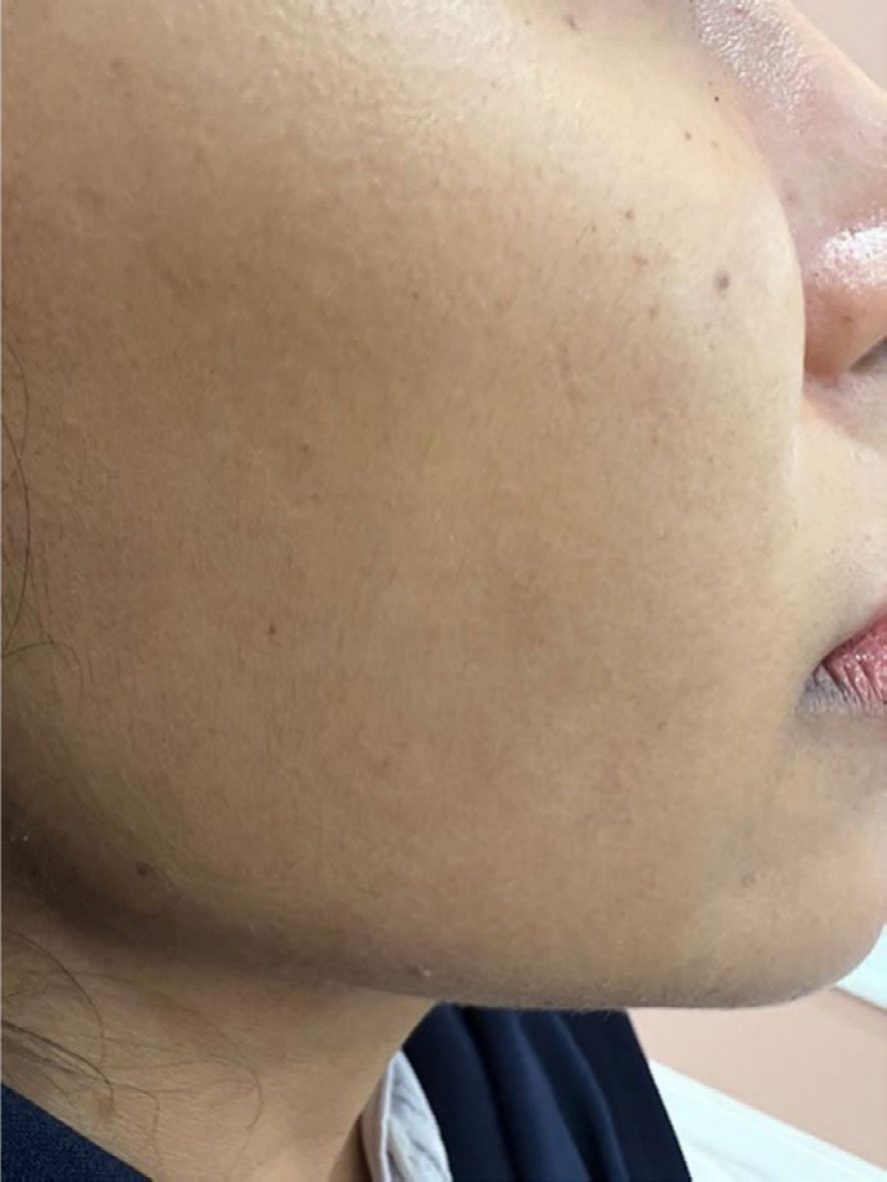
Same patient 16 days after intralesional hyaluronidase, showing marked resolution of the inflammatory submalar nodules.

A similar but less pronounced inflammatory reaction recurred following the patient's third dose of dupilumab on September 12, 2024. She returned to our clinic on September 19, 2024, with persistent bilateral malar nodules, again more prominent on the right side. Physical examination revealed firm, mildly tender nodules at the sites of the previous HA skinbooster injections, without overlying erythema, fluctuance, drainage, or other signs of infection. Additional hyaluronidase was administered bilaterally, resulting in further reduction in nodule size over the subsequent week (Figure 3). The Naranjo Adverse Drug Reaction Probability Scale score was 8, indicating a probable association between dupilumab administration and the delayed inflammatory reaction to the HA filler.

## 3 Discussion

With a Naranjo Adverse Drug Reaction Probability Scale score of 8 in this case, the correlation between dupilumab administration and the inflammatory filler reaction is probable but not definitive. It is pertinent to highlight that this score is not definite proof, but a probability based on clinical criteria. The case means a temporal relationship could be supported; however, exclusion of infection, biofilm formation, a spontaneous delayed filler reaction independent of dupilumab, and idiopathic granulomatous inflammation are not possible particularly in the absence of ultrasound imaging/biopsy/histopathological assessment/microbiologic culture.

Cross‐linked hyaluronic acid fillers are generally safe, with adverse effects consistent with those reported in prior clinical trials [7]. Delayed adverse events may arise from immune activation, bacterial contamination, biofilm formation, injection technique, or anatomical location [7]. The absence of confirmatory investigations in this case means that these alternative etiologies cannot be definitively ruled out.

Dupilumab is an IL‐4Rα inhibitor FDA‐approved for atopic dermatitis, CRSwNP, and asthma. It is generally well tolerated, with common side effects including injection‐site reactions and transient eosinophilia [8].

Dupilumab may have caused a delayed inflammatory reaction (DIR) at the locations of previous hyaluronic acid (HA) filler injection through a number of different mechanisms. The immunologic tolerance that had previously maintained a state of inactivity surrounding residual HA filler material was disturbed by dupilumab‐mediated suppression of IL‐4 and IL‐13, which is the most acceptable hypothesis. Important type 2 immunity regulators, IL‐4 and IL‐13, block Th1‐ and Th17‐mediated inflammatory pathways while promoting Th2 polarization and alternative (M2) macrophage activation [9, 10]. Dupilumab may eliminate this regulatory constraint and change the local immune environment to a Th1/Th17‐predominant state with increased production of proinflammatory cytokines, such as interferon‐γ, interleukin‐17, and tumor necrosis factor‐α, and classical (M1) macrophage activation [9, 10].

Remaining HA filler and its breakdown products may act as endogenous danger‐associated molecular patterns (DAMPs) and persistent foreign‐body substrates in this altered immune setting. These pathways can activate innate immune receptors like CD44 and Toll‐like receptors 2 and 4.2, 5, 7, which may increase macrophage recruitment and cytokine release, turning previously asymptomatic filler deposits into localized foci of inflammation and nodule formation. The idea that dupilumab‐induced immune reprogramming revealed a localized macrophage‐mediated foreign‐body inflammatory response to residual HA filler material is supported by the repeatable recurrence of the reaction following each dupilumab dose and the consistent clinical improvement following enzymatic dissolution of the filler with hyaluronidase. However, due to the lack of histological and microbiological proof, this suggested mechanism is still theoretical. Accordingly, granulomatous inflammation should not be regarded as a definitive diagnosis in this case.

Since removing the filler substrate was thought to be the most straightforward way to get rid of the hypothesized source of the inflammatory reaction, hyaluronidase was chosen as the main therapy. Due to diagnostic uncertainty, systemic corticosteroids were considered but withheld. Antibiotics were not provided because there were no clinical signs of infection, and antihistamines were not given since the clinical presentation did not match an IgE‐mediated hypersensitivity reaction. However, it should be mentioned that corticosteroids continue to be the preferred therapy for granulomatous delayed filler responses and histologically verified type IV hypersensitivity reactions, while antihistamines are often ineffectual in these situations [11].

To our knowledge, no published case reports have linked biologic therapy to inflammatory reactions involving previously placed hyaluronic acid (HA) fillers. However, comparable reactions have been documented following exposure to systemic immune stimuli like Zoledronic acid and Shingrix and Fluzone vaccinations, indicating that in susceptible individuals, systemic immune modulation may reactivate inflammatory responses at sites containing residual filler material [12, 13].

This case report has several important limitations. The absence of ultrasound imaging, tissue biopsy, histopathological examination, and microbiological culture substantially limits diagnostic certainty and represents the principal constraint on interpretation. Without histopathological confirmation, the diagnosis of a dupilumab‐associated inflammatory filler reaction remains presumptive, and alternative explanations—including infection, bacterial biofilm formation, and idiopathic granulomatous inflammation—cannot be definitively excluded. Future reports of suspected biologic‐associated filler reactions should, whenever feasible and with patient consent, incorporate ultrasound‐guided evaluation and tissue sampling to better characterize the underlying pathology. In addition, the Naranjo Adverse Drug Reaction Probability Scale provides only a probabilistic estimate of causality based on clinical criteria and does not establish a direct mechanistic relationship. Accordingly, the findings should be interpreted as hypothesis‐generating and with appropriate caution.

## 4 Conclusion

Delayed inflammatory reactions to HA fillers may develop months after the original injection, presenting as firm, localized nodules at prior injection sites. In this case, reactions appeared to be triggered by dupilumab administration, with recurrence following each subsequent dose. To our knowledge, this is the first published case report describing this specific interaction. The evidence in this case is constrained by the absence of imaging and histopathological investigation, and the causal relationship between dupilumab and the observed reactions remains probable rather than confirmed. Infection, biofilm, and idiopathic granulomatous inflammation cannot be excluded. Despite the limitations of our study, this case highlights a need for increased clinician awareness and consideration of potential interaction between biologic therapies and previously placed HA fillers. Patients receiving immunomodulatory treatments with a history of HA filler injections should be counseled on such rare potential risks. Prospective studies elucidating the immunological underpinning of this interaction and mechanistic studies are needed to inform evidence‐based management recommendations.

## Funding

The authors have nothing to report.

## Ethics Statement

The authors have nothing to report.

## Consent

Written informed consent was obtained from the patient for publication of clinical information and accompanying images. All reasonable efforts have been made to protect the patient's identity; however, anonymity cannot be guaranteed.

## Conflicts of Interest

The authors declare no conflicts of interest.

## Data Availability

The data that support the findings of this study are available from the corresponding author upon reasonable request.

## References

[1] American Society of Plastic Surgeons , 2022 Plastic Surgery Statistics Report (American Society of Plastic Surgeons, 2023).

[2] S. P. Fundarò , G. Salti , D. M. H. Malgapo , et al., “The Rheology and Physicochemical Characteristics of Hyaluronic Acid Fillers: Their Clinical Implications,” International Journal of Molecular Sciences 23, no. 18 (2022): 10518, 10.3390/ijms231810518. 36142430 PMC9503994

[3] O. Alkhuzaim , N. L. Roswandi , M. Waqas , et al., “Hyaluronic Acid, a Promising Skin Rejuvenating Biomedicine: A Review of Recent Updates and Pre‐Clinical and Clinical Investigations on Cosmetic and Nutricosmetic Effects,” International Journal of Biological Macromolecules 120, no. Pt B (2018): 1682–1695, 10.1016/j.ijbiomac.2018.09.188. 30287361

[4] I. Bogdan Allemann and L. Baumann , “Hyaluronic Acid Gel (Juvéderm) Preparations in the Treatment of Facial Wrinkles and Folds,” Clinical Interventions in Aging 3, no. 4 (2008): 629–634, 10.2147/cia.s3118. 19281055 PMC2682392

[5] M. G. Turkmani , K. De Boulle , and W. G. Philipp‐Dormston , “Delayed Hypersensitivity Reaction to Hyaluronic Acid Dermal Filler Following Influenza‐Like Illness,” Clinical, Cosmetic and Investigational Dermatology 12 (2019): 277–283, 10.2147/CCID.S198081. 31118731 PMC6501047

[6] C. Bachert , J. K. Han , M. Desrosiers , et al., “Efficacy and Safety of Dupilumab Patients With Severe Chronic Rhinosinusitis With Nasal Polyps (LIBERTY NP SINUS‐24 and LIBERTY NP SINUS‐52): Results From Two Multicentre, Randomised, Double‐Blind, Placebo‐Controlled, Parallel‐Group Phase 3 Trials,” Lancet 394, no. 10209 (2019): 1638–1650, 10.1016/S0140-6736(19)31881-1. 31543428

[7] O. Alkhuzaim , “Dermal Fillers in Aesthetics: An Overview of Adverse Events and Treatment Approaches,” Clinical, Cosmetic and Investigational Dermatology 6 (2013): 295–316, 10.2147/CCID.S50546. 24363560 PMC3865975

[8] A. Wollenberg , L. A. Beck , A. Blauvelt , et al., “Laboratory Safety of Dupilumab in Moderate‐to‐Severe Atopic Dermatitis: Results From Three Phase III Trials,” British Journal of Dermatology 182, no. 5 (2020): 1120–1135, 10.1111/bjd.18434. 31407311 PMC7317598

[9] N. A. Gandhi , B. L. Bennett , N. M. H. Graham , et al., “Targeting Key Proximal Drivers of Type 2 Inflammation in Disease,” Nature Reviews Drug Discovery 15, no. 1 (2016): 35–50, 10.1038/nrd4624. 26471366

[10] N. A. Gandhi , G. Pirozzi , and N. M. H. Graham , “Commonality of the IL‐4/IL‐13 Pathway in Atopic Diseases,” Expert Review of Clinical Immunology 13, no. 5 (2017): 425–437, 10.1080/1744666X.2017.1298443. 28277826

[11] W. Baranska‐Rybak , J. V. Lajo‐Plaza , L. Walker , et al., “Late‐Onset Reactions After Hyaluronic Acid Dermal Fillers: A Consensus Recommendation on Etiology, Prevention and Management,” Dermatology and Therapy 14, no. 7 (2024): 1767–1785, 10.1007/s13555-024-01202-3. 38907876 PMC11265052

[12] N. J. Alghamdi , S. R. Almuhaidib , A. S. Alharbi , et al., “Delayed Inflammatory Reaction to Hyaluronic Acid Dermal Filler Following Zoledronic Acid Administration: A Case Report,” Clinical, Cosmetic and Investigational Dermatology 17 (2024): 1347–1350, 10.2147/CCID.S458750. 38895606 PMC11182873

[13] J. Witt , D. Hooper , and G. G. Munavalli , “Delayed Inflammatory Reaction to Hyaluronic Acid Filler Following Shingrix and Fluzone Vaccines Treated With Lisinopril,” JAAD Case Reports 23 (2022): 133–135, 10.1016/j.jdcr.2022.03.018. 35495972 PMC9052063

